# Diagnostic accuracy of postmortem ultrasound *vs* postmortem 1.5‐T MRI for non‐invasive perinatal autopsy

**DOI:** 10.1002/uog.22012

**Published:** 2021-03-01

**Authors:** S. C. Shelmerdine, N. J. Sebire, O. J. Arthurs

**Affiliations:** ^1^ Department of Clinical Radiology Great Ormond Street Hospital for Children London UK; ^2^ UCL Great Ormond Street Institute of Child Health Great Ormond Street Hospital for Children London UK; ^3^ Department of Histopathology Great Ormond Street Hospital for Children London UK; ^4^ NIHR Great Ormond Street Hospital Biomedical Research Centre London UK

**Keywords:** autopsy, magnetic resonance imaging, pediatric, perinatal, ultrasound

## Abstract

**Objectives:**

To determine the diagnostic accuracy of postmortem magnetic resonance imaging (PM‐MRI) and postmortem ultrasound (PM‐US) for perinatal autopsy in the same patient cohort, and to determine whether PM‐US can provide the same anatomical information as PM‐MRI.

**Methods:**

In this prospective, 5‐year (July 2014–July 2019) single‐center study, we performed 1.5‐T PM‐MRI and PM‐US in an unselected cohort of perinatal deaths. The diagnostic accuracies of both modalities were calculated, using autopsy as the reference standard. As a secondary objective, the concordance rates between the two imaging modalities for the overall main diagnosis and for five anatomical regions (brain, spine, thorax, heart and abdomen) were calculated.

**Results:**

During the study period, 136 cases underwent both PM‐US and PM‐MRI, of which 88 (64.7%) also underwent autopsy. There was no significant difference in the rates of concordance with autopsy between the two modalities for overall diagnosis (PM‐US, 86.4% (95% CI, 77.7–92.0%) *vs* PM‐MRI, 88.6% (95% CI, 80.3–93.7%)) or in the sensitivities and specificities for individual anatomical regions. There were more non‐diagnostic PM‐US than PM‐MRI examinations for the brain (22.8% *vs* 3.7%) and heart (14.7% *vs* 5.1%). If an ‘imaging‐only’ autopsy had been performed, PM‐US would have achieved the same diagnosis as 1.5‐T PM‐MRI in 86.8% (95% CI, 80.0–91.5%) of cases, with the highest rates of agreement being for spine (99.3% (95% CI, 95.9–99.9%)) and cardiac (97.3% (95% CI, 92.4–99.1%)) findings and the lowest being for brain diagnoses (85.2% (95% CI, 76.9–90.8%)).

**Conclusion:**

Although there were fewer non‐diagnostic cases using PM‐MRI than for PM‐US, the high concordance rate for overall diagnosis suggests that PM‐US could be used for triaging cases when PM‐MRI access is limited or unavailable. © 2020 The Authors. Ultrasound in Obstetrics & Gynecology published by John Wiley & Sons Ltd on behalf of International Society of Ultrasound in Obstetrics and Gynecology.

**CONTRIBUTION**

*What are the novel findings of this work?*

There are no statistically significant differences in diagnostic accuracy compared with autopsy between postmortem ultrasound (PM‐US) and postmortem 1.5‐T magnetic resonance imaging (PM‐MRI) for non‐invasive perinatal autopsy. Non‐diagnostic imaging was more likely on ultrasound than on 1.5‐T MRI, particularly for the brain and heart.

*What are the clinical implications of this work?*

When there is limited or no access to PM‐MRI, PM‐US imaging is a viable alternative. It is a suitable modality for centers wishing to offer non‐invasive imaging alternatives to perinatal autopsy.

## INTRODUCTION

A perinatal autopsy can provide useful additional clinical information in approximately 25–36% of cases^1^, ^2^, not only allowing parents to understand the circumstances surrounding their child's death, but also helping to refine clinical management for future pregnancies. Nevertheless, more than half of all parents typically decline a conventional autopsy for personal, emotional or religious reasons, many referring to the invasive nature of the procedure^3^, ^4^, ^5^. Non‐invasive autopsies, utilizing imaging techniques, have therefore seen an increase in popularity, with postmortem magnetic resonance imaging (PM‐MRI) and postmortem ultrasound (PM‐US) demonstrating high rates of concordance with autopsy as the reference standard^6^, ^7^.

Much of the published literature relating to PM‐US and PM‐MRI reports diagnostic accuracy rates within different cohorts of patients, making direct comparison of these two imaging modalities difficult. Recent work by Kang *et al*.^8^ showed that when PM‐US and PM‐MRI (at 3T) were used in the same cohort of fetuses which underwent perinatal death, their rates of concordance with autopsy were similar when diagnostic‐quality imaging was achieved (approximately 81–96% concordance with autopsy). However, access to 3‐T MRI may be limited and, while 1.5‐T MRI is much more widely available, some centers may have no practical access to MRI at all, as clinical scanner time may mean that postmortem cases cannot be accommodated. How the diagnostic accuracy compares between PM‐US and 1.5‐T PM‐MRI in the same perinatal cohort is currently unknown. This, however, is important, as PM‐US could be an easily accessible, cheaply available alternative screening tool when access to MRI is limited, providing it has sufficient diagnostic accuracy.

In this prospective, single‐center cohort study, our objectives were two‐fold. First, we aimed to determine the diagnostic accuracy of both 1.5‐T PM‐MRI and PM‐US, using autopsy as the reference standard, in the same cohort of perinatal deaths. Second, we aimed to review the major differences between the two imaging modalities in overall and organ‐specific diagnosis, to evaluate the impact that using PM‐US rather than 1.5‐T PM‐MRI would have. These outcomes could provide an evidence basis for setting national non‐invasive autopsy imaging protocols.

## METHODS

Ethical approval was granted for this single‐center, prospective cohort study, conducted at Great Ormond Street Hospital, London, UK (IRAS ID:13195; REC reference: 13/LO/1494). All parents gave written consent allowing postmortem imaging and autopsy (where this was performed) to be conducted. The study was approved by a national research ethics committee (REC 09/H0713/2) and all samples were handled in accordance with the Human Tissue Act (2004).

### Patient selection

We included in this study, over a 5‐year period (July 2014–July 2019), consecutive unselected perinatal deaths which had both perinatal PM‐US and 1.5‐T PM‐MRI. Cases referred for any type of perinatal autopsy were included in our cohort. The decision to undertake both imaging modalities was not predetermined, being based on the availability of both PM‐US and 1.5‐T PM‐MRI at the time of case referral, and was performed in an arbitrary order. No inclusion or exclusion criteria were applied.

Demographic details for each case, including gestational age, gender, mode of fetal death, date of birth/death, postmortem weight and fetal size (i.e. crown–rump and crown–heel lengths) were documented. A maceration score (based on a severity scale of 0 to 3; with 3 assigned to cases with extensive/marked maceration) was also assigned to each case, based on the pathologists' description at external examination.

### PM‐MRI and PM‐US imaging and reporting

PM‐MRI was performed on a 1.5‐T Avanto (Siemens, Munich, Germany) scanner, according to published local departmental protocols^9^, ^10^. In brief, this included whole‐body, isovolumetric T2‐weighted and T1‐weighted sequences with diffusion‐weighted imaging. PM‐US examinations were performed using a dedicated ultrasound machine based in the hospital mortuary (UGEO HM70A, Samsung, Munich, Germany, equipped with a 7–16‐MHz linear probe). The examinations were performed by a pediatric radiology research fellow (S.C.S., with 4 years' experience in postmortem pediatric imaging and 6 years' general pediatric radiology experience) according to previous publications^11^, ^12^.

The radiology report was issued on the same day as the imaging examination, and entered into our local radiology information system and the pathology database. All radiology reports followed a predefined reporting template, divided according to five anatomical regions: brain, spine, heart, thorax and abdomen. For each area, the radiologist specified either ‘normal’ or ‘abnormal’, with further description of the particular abnormality and organ involved within the body area. A final overall diagnosis (normal/abnormal, with further description) was also provided. When the imaging for a particular anatomical region was non‐diagnostic, this was recorded also.

The PM‐MRI findings were reported by a specialist pediatric radiologist with expertise in postmortem imaging (O.J.A., with 10 years' experience of postmortem pediatric imaging). The PM‐US studies were reported by the same radiology research fellow who had performed the examination (S.C.S.). Both radiologists were blinded to the antenatal and maternal history, and to each other's reports; they were informed only of the patient's gestational age and manner of death (i.e. termination of pregnancy, stillbirth, miscarriage).

### Histological sampling and autopsy

When parental consent had been provided for a full, conventional autopsy, this was conducted according to the Royal College of Pathologists autopsy guidelines^13^, ^14^, ^15^ by one of seven experienced pediatric pathologists at our institution. When consent had been provided for a minimally invasive autopsy (MIA), this was either conducted by a pediatric pathologist via a laparoscopic keyhole technique^16^, ^17^ or the radiology research fellow performed ultrasound‐guided biopsies^18^ of the major organs, which were then assessed histologically by a pediatric pathologist. When consent was given only for external, non‐invasive invasive autopsy, no incisions were made or tissue sampling performed. As part of routine practice, dissection of the spine is not performed for any of the autopsy types. For MIA, brain dissection is not performed unless there is specific additional consent for this.

In all cases, the pediatric pathologist issuing the final autopsy report was aware of the imaging findings. The results from the autopsy were subsequently entered into the pathology database, with abnormalities being specified according to the same five predefined body areas as in the imaging reports.

### Data analysis

Results from the radiology and autopsy reports were extracted from the pathology database and input into a dedicated research database in Microsoft Excel (Microsoft Corp., Redmond, WA, USA). For our primary aim, we calculated the diagnostic accuracy rates for PM‐MRI and PM‐US, using autopsy as the reference standard. In this calculation, we included only cases in which histological tissue sampling had been performed (i.e. full conventional autopsy or MIA). Descriptive statistics and diagnostic accuracy calculations using exact methods were used to derive sensitivity, specificity, positive (PPV) and negative (NPV) predictive values and positive and negative likelihood ratios, as well as concordance for organ‐specific findings and overall diagnoses. Non‐diagnostic body parts were also analyzed, but excluded from accuracy rate calculations. For our secondary aim, all cases, which had undergone both PM‐US and PM‐MRI (regardless of autopsy type), were included for analysis and overall differences between them with respect to both the main diagnosis and the findings according to organ system were compared using descriptive statistics.

### Statistical power calculation

Prior to the study, we calculated the sample size required to detect a 10% difference in concordance between PM‐MRI and PM‐US in all five anatomical regions assuming independence between regions, a 5% significance level and 90% power. The sample‐size formula for a matched comparison of two diagnostic tests was used^19^. Calculations were based on concordance estimates derived from local PM‐MRI data^6^ and assumed that PM‐US has 10% lower sensitivity in all five anatomical areas. The abdominal region required the largest sample size to maintain 90% power. Assuming an MRI concordance with autopsy of 89% in the abdominal region (and therefore 79% for PM‐US), we determined that a cohort of at least 278 cases would be required.

## RESULTS

### Study cohort

In total, over the study period, 136 fetuses underwent perinatal death and had both PM‐US and 1.5‐T PM‐MRI. The population demographics are summarized in Table 1. Of these, 88 (64.7%) underwent autopsy that included histological tissue sampling (32/88 (36.4%) conventional autopsy and 56/88 (63.6%) MIA), and 31/88 (35.2%) had neuropathological examination with brain dissection and histology.

**Table 1:** Demographics of study cohort of perinatal deaths, overall and according to type of autopsy

		Autopsy type		
Parameter	Whole cohort (*n* = 136)	Conventional (*n* = 32)	Minimally invasive*(*n* = 56)	Non‐invasive (*n* = 48)
Gender				
Male	82 (60.3)	19 (59.4)	30 (53.6)	33 (68.8)
Female	54 (39.7)	13 (40.6)	26 (46.4)	15 (31.3)
Mode of death				
TOP	14 (10.3)	23 (71.9)	14 (25.0)	18 (37.5)
Miscarriage	30 (22.1)	1 (3.1)	13 (23.2)	16 (33.3)
IUFD/stillbirth	90 (66.2)	7 (21.9)	29 (51.8)	13 (27.1)
NND	2 (1.5)	1 (3.1)	0	1 (2.1)
Maceration severity				
None	52 (38.2)	12 (37.5)	15 (26.8)	25 (52.1)
Mild	31 (22.8)	8 (25.0)	13 (23.2)	10 (20.8)
Moderate	15 (11.0)	6 (18.8)	3 (5.4)	6 (12.5)
Extensive/marked	38 (27.9)	6 (18.8)	25 (44.6)	7 (14.6)
Gestational age at delivery (weeks) (*n* = 134 fetuses)	27 (15–42)	26 (15–42)	29 (17–42)	24 (15–39)
Age at PM (days) (*n* = 2 NND)	11.5 (4–19)	19	N/A	4
PM weight (g)	1127 (56–4060)	1091 (56–3320)	1391 (63–4060)	843 (85–3324)
Crown–rump length (cm)	23 (7–38)	23 (10–36)	25 (7–38)	21 (12–36)
Crown–heel length (cm)	33 (10–55)	33 (14–51)	35 (10–55)	30 (16–52)
Time from delivery to PM‐MRI (days)	10 (0–35)	8 (3–35)	10 (4–35)	10 (0–19)
Time from delivery to PM‐US (days)	10 (0–41)	8 (0–39)	12 (3–41)	9 (1–21)
Time between PM‐MRI and PM‐US (days)	2 (0–13)	2 (0–9)	3 (0–9)	3 (0–13)
Time from delivery to autopsy (days)	12 (4–47)	11 (4–47)	12 (4–42)	N/A

Data are given as *n* (%) or mean (range).

* Laparoscopic‐guided biopsy.

IUFD, intrauterine fetal death; MRI, magnetic resonance imaging; N/A, not applicable; NND, neonatal death; PM, postmortem; TOP, termination of pregnancy; US, ultrasound.

Cases that did not have an autopsy were of a slightly lower average gestational age (24 weeks (non‐invasive autopsy) *vs* 26 weeks (full, conventional autopsy) and 29 weeks (MIA)), and thus had an associated lower postmortem weight and length. Cases that underwent MIA were more likely to be markedly macerated (44.6% of cases) than were those which underwent full autopsy (18.8%) or non‐invasive autopsy (14.6%).

### Diagnostic accuracy of PM‐US and PM‐MRI (compared with autopsy)

Among the 88 cases which underwent autopsy that included histological tissue sampling, for overall main diagnosis, there was no statistical difference between the two postmortem imaging modalities in sensitivity (78.0% (95% CI, 63.3–88.0%) for PM‐US *vs* 90.2% (95% CI, 77.5–96.1%) for PM‐MRI) or specificity (93.6% (95% CI, 82.8–97.8%) for PM‐US *vs* 87.2% (95% CI, 74.8–94.0%) for PM‐MRI) (Table 2). The greatest differences in sensitivity between the two modalities were for thoracic (40.0% (95% CI, 19.8–64.3%) for PM‐US *vs* 73.3% (95% CI, 48.0–89.1%) for PM‐MRI) and cardiac (50% (95% CI, 21.5–78.5%) for PM‐US *vs* 81.8% (95% CI, 52.3–94.9%) for PM‐MRI) imaging, but these did not reach statistical significance in our cohort.

**Table 2:** Postmortem ultrasound (PM‐US) and magnetic resonance imaging (PM‐MRI) diagnostic accuracy for individual body systems, all body systems summated and overall diagnoses, using autopsy as reference standard

	TP	FP	FN	TN	ND imaging	ND autopsy	No imaging	No autopsy	Sensitivity (%)	Specificity (%)	PPV (%)	NPV (%)	Concordance (%)
Brain PM‐US	7	0	1	11	31	5	3	105	87.5	100	100	91.7	94.7
									(52.9–97.8)	(74.1–100)	(64.6–100)	(64.6–98.5)	(75.4–99.1)
Brain	10	0	2	14	5	5	0	105	83.3	100	100	87.5	92.3
PM‐MRI									(55.2–95.3)	(78.5–100)	(72.2–100)	(64.0–96.5)	(75.9–97.9)
Cardiac	4	1	4	64	20	3	0	48	50.0	98.5	80.0	94.1	93.2
PM‐US									(21.5–78.5)	(91.8–99.7)	(37.6–96.4)	(85.8–97.7)	(84.9–97.0)
Cardiac	9	1	2	69	7	3	1	48	81.8	98.6	90.0	97.2	96.3
PM‐MRI									(52.3–94.9)	(92.3–99.7)	(59.6–98.2)	(90.3–99.2)	(89.7–98.7)
Thoracic	6	1	9	71	0	1	0	48	40.0	98.6	85.7	88.8	88.5
PM‐US									(19.8–64.3)	(92.5–99.8)	(48.7–97.4)	(80.0–94.0)	(80.1–93.6)
Thoracic	11	3	4	68	0	1	1	48	73.3	95.8	78.6	94.4	91.9
PM‐MRI									(48.0–89.1)	(88.3–98.6)	(52.4–92.4)	(86.6–97.8)	(84.1–96.0)
Abdominal	14	3	0	68	0	3	0	48	100	95.8	82.4	100	96.5
PM‐US									(78.5–100)	(88.3–98.6)	(59.0–93.8)	(94.7–100)	(90.1–98.8)
Abdominal	14	5	0	65	1	3	1	48	100	92.9	73.7	100	94.0
PM‐MRI									(78.5–100)	(84.3–96.9)	(51.2–88.2)	(94.4–100)	(86.8–97.4)
Total body	31	5	14	214	51	12	3	249	68.9	97.7	86.1	93.9	92.8
systems PM‐US									(54.3–80.5)	(94.8–99.3)	(71.3–93.9)	(90.0–96.3)	(89.0–95.3)
Total body	44	9	8	216	13	12	3	249	84.6	96.0	83.0	96.4	93.9
systems PM‐MRI									(72.5–92.0)	(92.6–97.9)	(70.8–90.8)	(93.1–98.2)	(90.4–96.1)
Overall	32	3	9	44	0	0	0	48	78.0	93.6	91.4	83.0	86.4
diagnosis* PM‐US									(63.3–88.0)	(82.8–97.8)	(77.6–97.0)	(70.8–90.8)	(77.7–92.0)
Overall	37	6	4	41	0	0	0	48	90.2	87.2	86.0	91.1	88.6
diagnosis* PM‐MRI									(77.5–96.1)	(74.8–94.0)	(72.7–93.4)	(79.3–96.5)	(80.3–93.7)

Values in parentheses are 95% CI.

There were no statistically significant differences in diagnostic accuracy between two imaging modalities.

*Overall diagnosis refers to major pathology identified as cause of perinatal death.

FN, false negative; FP, false positive; ND, non‐diagnostic; NPV, negative predictive value; PPV, positive predictive value; TN, true negative; TP, true positive.

In Table S1 we have provided the positive and negative likelihood ratios of both imaging modalities for the different body systems and the overall diagnosis. The highest positive likelihood ratios were for brain PM‐MRI and PM‐US diagnoses (∞), with those for overall diagnosis on PM‐US being 12.23 (95% CI, 4.04–36.99) and on PM‐MRI being 7.07 (95% CI, 3.33–15.03). The values for both positive and negative likelihood ratios were not significantly different between PM‐US and PM‐MRI.

Details of individual false‐negative and false‐positive diagnoses are provided according to anatomical region in Table S2. For the brain, there were no false‐positive diagnoses generated by either imaging modality, although PM‐MRI failed to identify one case of cerebellar hypoplasia and both modalities were unable to detect a case of severe hypoxic‐ischemic encephalopathy with periventricular necrosis and hindbrain neuronal loss. Discrepancies in thoracic findings arose mainly from the overcalling or missing of pulmonary hypoplasia (in three cases for PM‐US only; in two cases for PM‐MRI only; and in three cases for both modalities), suggesting a high level of subjective opinion in this diagnosis. For cardiac anomalies, PM‐US missed four diagnoses (one case of dilated cardiomyopathy, one of double‐outlet right ventricle (DORV), one of cardiac hypertrophy and dysplastic pulmonary valve, and one, also missed by PM‐MRI, of cardiomegaly) and overcalled one case of ventricular septal defect (VSD), while PM‐MRI missed two cardiac anomalies (the case of cardiomegaly and one of VSD) and overcalled one case of DORV.

There were no misses on either modality for abdominal pathologies. However, PM‐MRI generated more false‐positive diagnoses compared with PM‐US; two abnormalities were overcalled on both imaging modalities, one case of anal atresia (in which the anus was present and patent) and one case of intra‐abdominal gas with suspected sepsis (in which no organisms were identified on microbiology of the abdomen, although the placenta demonstrated chorioamnionitis).

**Figure 1:**
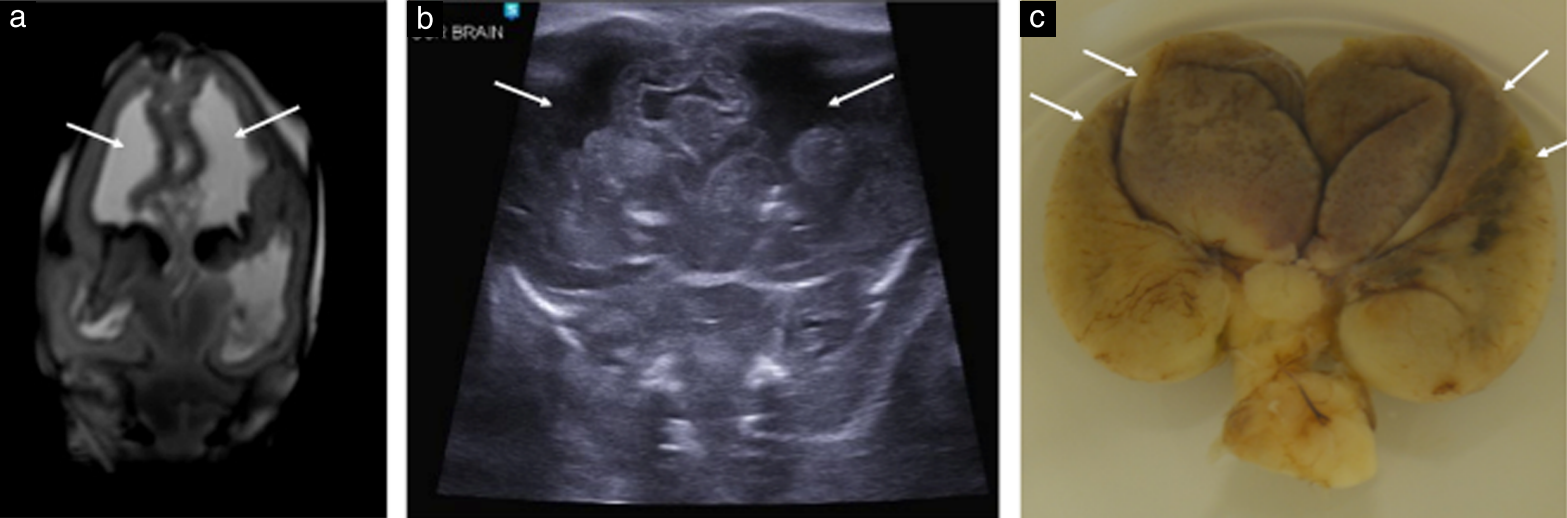
Postmortem imaging in 18‐week fetus with ventriculomegaly, after termination of pregnancy: example of concordant diagnosis between postmortem ultrasound (PM‐US) and postmortem magnetic resonance imaging (PM‐MRI), with autopsy confirmation. (a) Coronal T2‐weighted PM‐MRI image through foramen of Monroe, demonstrating bilateral ventriculomegaly (arrows). (b) Corresponding coronal image of brain on PM‐US demonstrating ventriculomegaly (arrows). (c) Macroscopic photograph of extracted brain, taken in water bath at autopsy, demonstrating dilated, ‘baggy’‐appearing cerebral hemispheres (arrows) in keeping with underlying ventricular dilatation.

**Figure 2:**
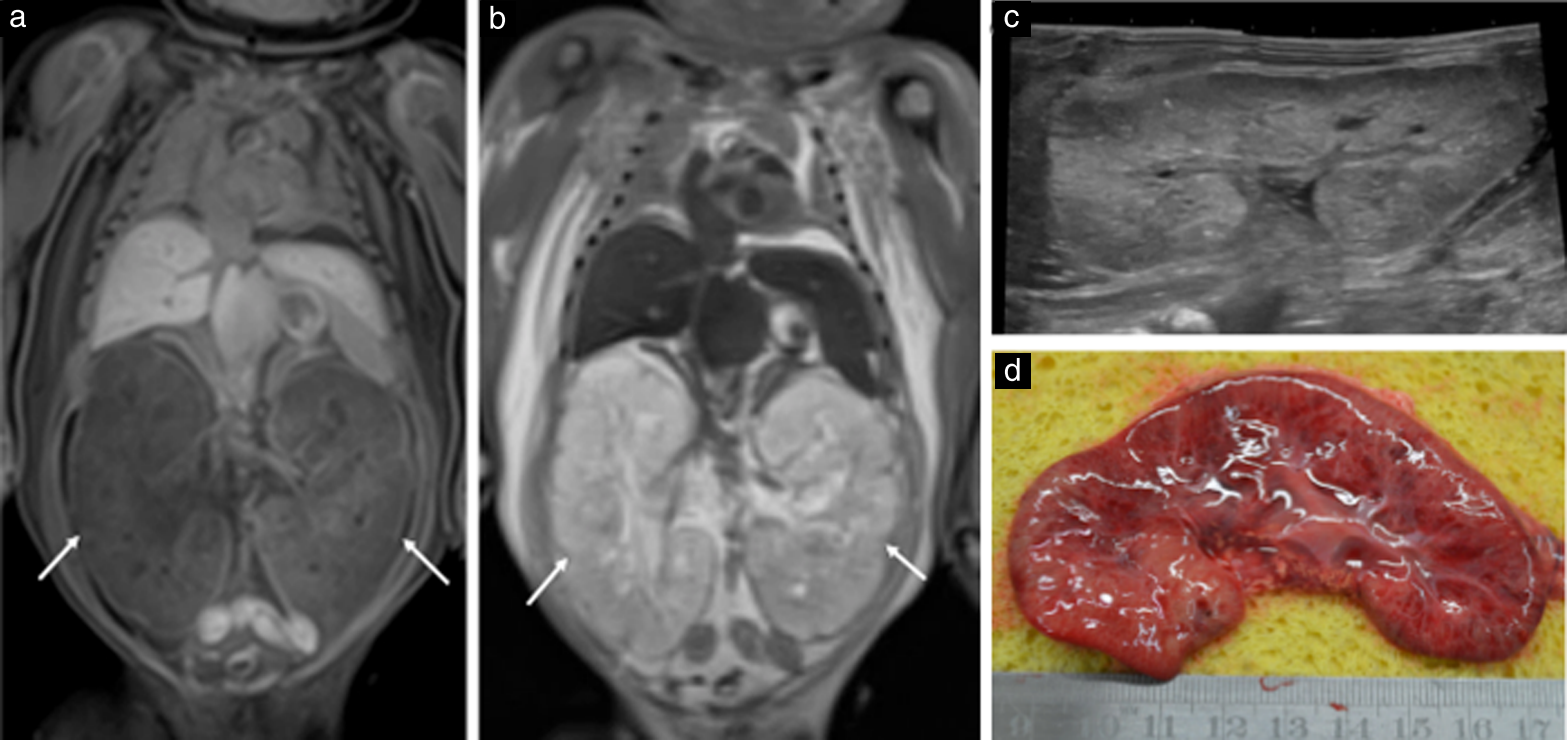
Postmortem imaging of bilateral, enlarged polycystic kidneys in stillborn 33‐week fetus with autosomal recessive polycystic kidney disease: example of concordant diagnosis between postmortem ultrasound (PM‐US) and postmortem magnetic resonance imaging (PM‐MRI), with autopsy confirmation. (a,b) Coronal T1‐weighted (a) and T2‐weighted (b) PM‐MRI images of body, demonstrating bilateral enlarged kidneys (arrows) with internal small cysts. (c) Sagittal PM‐US image of left kidney demonstrating enlarged, echogenic kidney, in keeping with multiple microscopic cysts in kidney. (d) Macroscopic photograph of left kidney at autopsy, demonstrating appearance similar to that on imaging.

**Figure 3:**
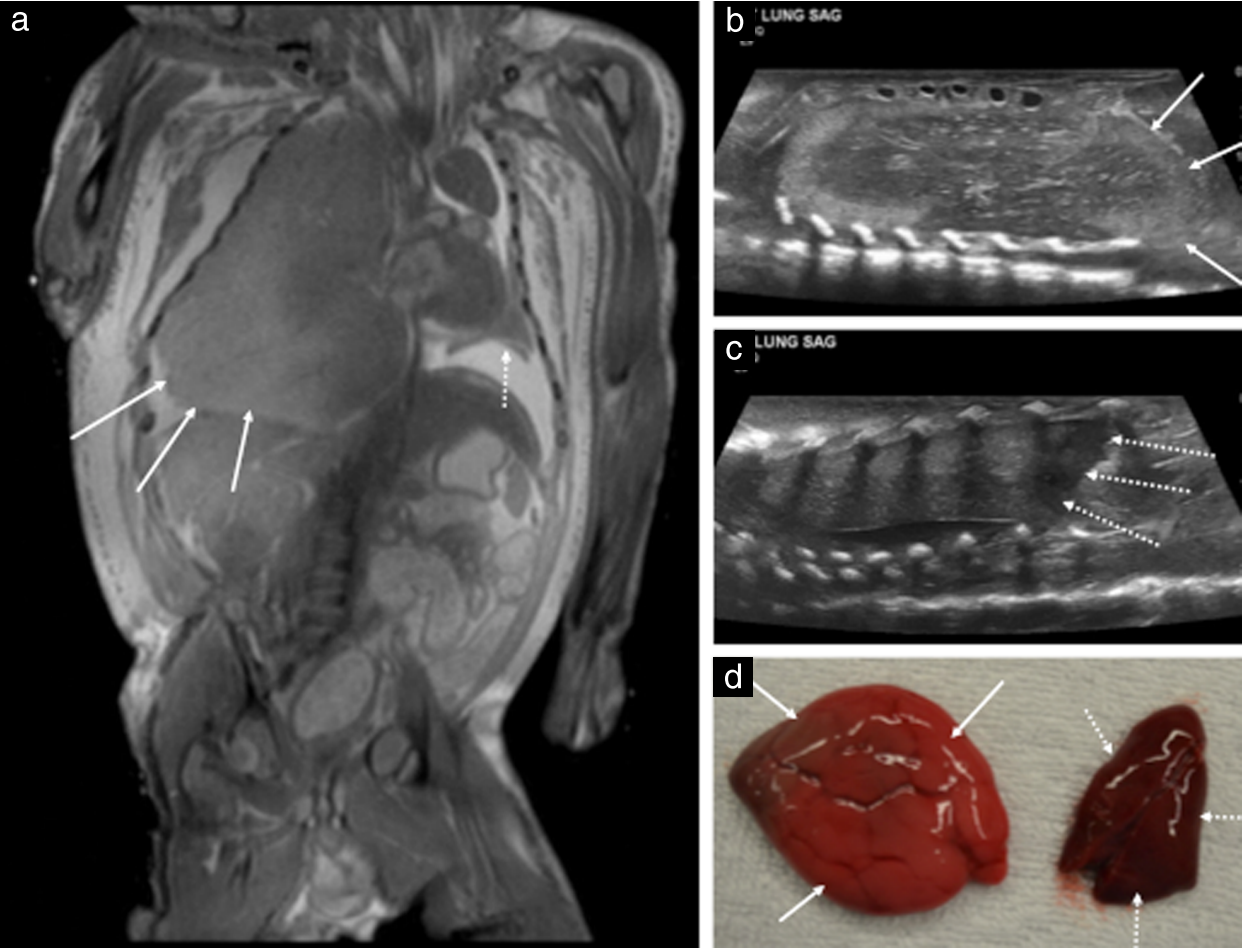
Postmortem imaging of right‐sided congenital lobar overinflation in 23‐week fetus: example of correct diagnosis on postmortem magnetic resonance imaging (PM‐MRI), but false negative (i.e. missed diagnosis) on postmortem ultrasound (PM‐US). (a) Coronal T2‐weighted PM‐MRI image of thorax and abdomen, demonstrating very enlarged right lung with bulging lower lobe (solid arrows) that displace inferiorly right hemidiaphragm. There is also mediastinal shift to left, with comparatively smaller left lung (dashed arrow). (b) Sagittal PM‐US image of right lung showing bulging inferior lobe (arrows) and heterogeneous internal lung parenchyma which was not reported or identified as abnormal at time of imaging. (c) Sagittal PM‐US image of left lung is provided for comparison, showing normal appearance of left hemidiaphragm (dashed arrows) with no overinflation of lung. (d) Macroscopic photographs of extracted lungs at autopsy, demonstrating differences in appearance between abnormal right lung (solid arrows) and normal left lung (dashed arrows).

**Figure 4:**
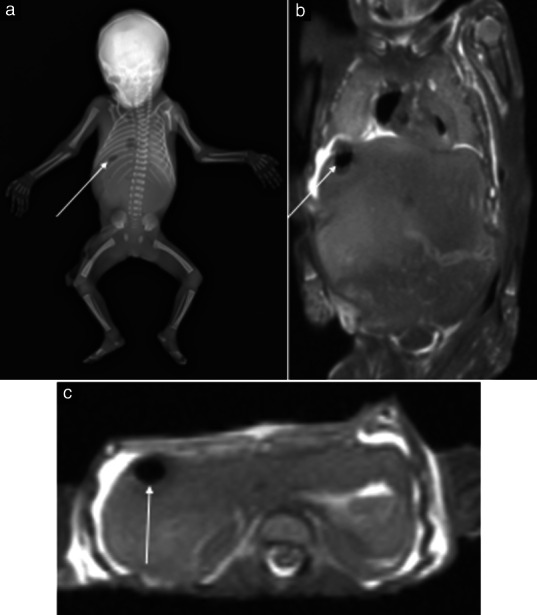
Normal postmortem appearance in miscarried 20‐week fetus which was diagnosed incorrectly with bowel perforation on postmortem magnetic resonance imaging (PM‐MRI). (a) Frontal view of whole‐body postmortem skeletal radiogram showing tiny locule of gas in right upper quadrant of abdomen (arrow). (b,c) Coronal (b) and axial (c) T2‐weighted PM‐MRI images of abdomen demonstrating small locule of gas (arrow); this was thought to represent bowel perforation, of which there was no evidence at autopsy.

**Figure 5:**
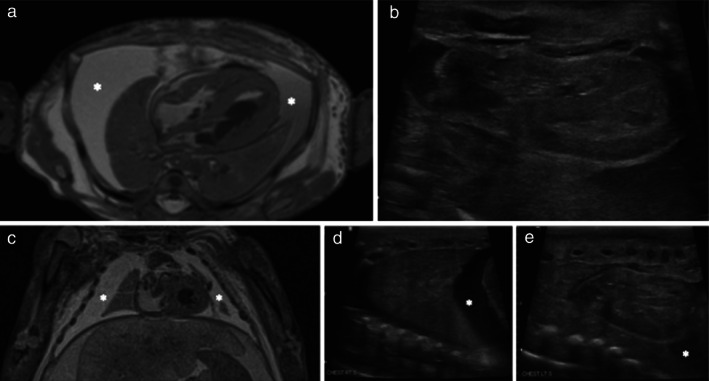
Postmortem imaging in 30‐week fetus with hydrops secondary to underlying cytomegalovirus infection. Neither postmortem ultrasound (PM‐US) nor postmortem magnetic resonance imaging (PM‐MRI) identified any infective process in lungs or presence of cardiomegaly, which was reported at autopsy. (a,b) Axial T2‐weighted PM‐MRI (a) and transverse PM‐US imaging (b) of heart failed to report cardiomegaly, presumably due to more striking appearance of large bilateral pleural effusions (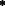). (c–e) Coronal T2‐weighted PM‐MRI (c) and sagittal PM‐US of the right (d) and left (e) lungs, showing bilateral pleural effusions (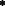) and typical appearance of consolidated lungs on postmortem imaging.

Examples of cases in which both imaging modalities identified correctly the pathological diagnosis are shown in Figures 1 and 2, while Figures 3, 4, 5 give examples of when one or both modalities were inaccurate.

#### Cases with autopsy, but no neuropathology

For the 57/88 (64.8%) cases in which the brain was not examined at autopsy, the breakdown of imaging results were as follows. On both PM‐US and PM‐MRI, 32 of the 57 (56.1%) were normal and 1/57 (1.8%) was judged to have absent corpus callosum (ACC), ventriculomegaly and periventricular nodular heterotopia. On PM‐MRI only (PM‐US was normal), 1/57 (1.8%) had isolated ACC, 1/57 (1.8%) had cystic hygroma and 1/57 (1.8%) had cerebellar hypoplasia and mega cisterna magna. There were no abnormalities observed only on PM‐US and not on PM‐MRI. In 16/57 (28.1%) cases, PM‐US was non‐diagnostic while PM‐MRI was normal, in 3/57 (5.3%) cases the brain was not examined at PM‐US due to overlapping sutures (PM‐MRI was normal) and in 2/57 (3.5%) both modalities were non‐diagnostic.

### Agreement between PM‐US and 1.5‐T PM‐MRI

If PM‐US imaging were to be performed for all perinatal deaths instead of PM‐MRI, the same overall diagnosis would be seen in 86.8% (95% CI, 80.0–91.5%) of cases, with the highest concordance rates being for spine (99.3% (95% CI, 95.9–99.9%)) and cardiac (97.3% (95% CI, 92.4–99.1%)) diagnoses and the lowest concordance rate being for brain diagnoses (85.2% (95% CI, 76.9 – 90.8%)). PM‐US detected one case of hypoplastic cerebellum and one case of pelvic kidney for which PM‐MRI was negative or non‐diagnostic. PM‐US failed to diagnose an anomaly identified by PM‐MRI in 16/136 (11.8%) cases, of which 10 (62.5%) were brain‐related. Individual overall diagnoses by each imaging modality are detailed in Table S3, with a more detailed breakdown of the findings according to anatomical region being provided in Table S4.

### Non‐diagnostic imaging

There were more non‐diagnostic PM‐US than non‐diagnostic PM‐MRI studies for the brain and heart. In total, 31/136 (22.8%) brain and 20/136 (14.7%) cardiac PM‐US examinations were non‐diagnostic compared with only 5/136 (3.7%) brain and 7/136 (5.1%) cardiac PM‐MRI examinations (Table 3). Of these, 4/136 (2.9%) brain and 4/136 (2.9%) cardiac examinations were non‐diagnostic for both modalities.

**Table 3:** Demographics for cases with non‐diagnostic (ND) postmortem ultrasound (PM‐US), postmortem magnetic resonance imaging (PM‐MRI) and autopsy (including invasive and minimally invasive procedures)

Parameter	ND PM‐US	ND PM‐MRI	ND PM‐MRI and ND PM‐US	ND autopsy
Brain	31/136 (22.8)	5/136 (3.7)	4/136 (2.9)	5/88 (5.7)
GA at delivery ≤ 20 weeks	2/31 (6.5)	2/5 (40.0)	1/4 (25.0)	2/5 (40.0)
GA at delivery > 20 weeks	29/31 (93.5)	3/5 (60.0)	3/4 (75.0)	3/5 (60.0)
No maceration	1/31 (3.2)	1/5 (20.0)	0	0
Mild maceration	6/31 (19.4)	0	0	1/5 (20.0)
Moderate maceration	4/31 (12.9)	0	0	1/5 (20.0)
Extensive maceration	20/31 (64.5)	4/5 (80.0)	3/4 (75.0)	3/5 (60.0)
PMI 0–7 days	7/31 (22.6)	1/5 (20.0)	1/4 (25.0)	1/5 (20.0)
PMI 8–14 days	18/31 (58.1)	3/5 (60.0)	2/4 (50.0)	3/5 (60.0)
PMI > 15 days	6/31 (19.4)	1/5 (20.0)	1/4 (25.0)	1/5 (20.0)
Heart	20/136 (14.7)	7/136 (5.1)	4/136 (2.9)	3/88 (3.4)
GA at delivery ≤ 20 weeks	4/20 (20.0)	5/7 (71.4)	2/4 (50.0)	1/3 (33.3)
GA at delivery > 20 weeks	16/20 (80.0)	2/7 (28.6)	2/4 (50.0)	2/3 (66.7)
No maceration	3/20 (15.0)	2/7 (28.6)	0	0
Mild maceration	3/20 (15.0)	2/7 (28.6)	0	0
Moderate maceration	2/20 (10.0)	0	0	0
Extensive maceration	11/20 (55.0)	4/7 (57.1)	4/4 (100)	3/3 (100)
PMI 0–7 days	7/20 (35.0)	1/7 (14.3)	1/4 (25.0)	0
PMI 8–14 days	10/20 (50.0)	4/7 (57.1)	1/4 (25.0)	3/3 (100)
PMI > 15 days	3/20 (15.0)	2/7 (28.6)	2/4 (50.0)	0
Thorax	0	0	0	1/88 (1.1)
GA at delivery ≤ 20 weeks	0	0	0	1/1 (100)
Extensive maceration	0	0	0	1/1 (100)
PMI 8–14 days	0	0	0	1/1 (100)
Abdomen	0	1/136 (0.7)	0	3/88 (3.4)
GA at delivery ≤ 20 weeks	0	1/1 (100)	0	1/3 (33.3)
GA at delivery > 20 weeks	0	0	0	2/3 (66.7)
No maceration	0	1/1 (100)	0	0
Extensive maceration	0	0	0	3/3 (100)
PMI 8–14 days	0	0	0	3/3 (100)
PMI > 15 days	0	1/1 (100)	0	0
Spine	0	0	0	N/A

Data are given as *n*/*N* (%).

GA, gestational age; N/A, not applicable; PMI, postmortem interval (time between demise and procedure).

Table 3 presents various factors associated with non‐diagnostic PM‐US and PM‐MRI examinations, with particular emphasis on gestational age at delivery, extent of maceration and postmortem interval (PMI). Cases that were non‐diagnostic for the brain at PM‐US and PM‐MRI (both individually and combined) were more likely to be > 20 weeks' gestation, to have suffered marked maceration‐related changes and to have a time interval between delivery and imaging of 8–14 days. Cases that were non‐diagnostic for the heart at PM‐US and PM‐MRI (individually) were more likely to have extensive maceration and to have a PMI of 8–14 days, while those that were non‐diagnostic for both PM‐US and PM‐MRI were more likely to have a PMI > 15 days. There were no non‐diagnostic imaging examinations for the thorax or spine. PM‐MRI was non‐diagnostic for one abdominal study in a fetus < 20 weeks' gestation, without any maceration, imaged > 15 days post‐delivery.

### Pathological yield in cases with non‐diagnostic imaging

There were six cases with non‐diagnostic PM‐US of the brain for which autopsy data were available; there were no abnormalities in the brain in three (50.0%) of these cases. The three with brain pathology included one case of ACC, one case of ACC with occipital polymicrogyria and one case of vein of Galen malformation. Similarly, autopsy data showed no abnormalities of the heart in 9/12 (75.0%) cases with non‐diagnostic PM‐US; the cardiac anomalies present in the other three cases included one case of hypoplastic aortic arch, one VSD and one transposition of the great arteries. There were no cases in which there was non‐diagnostic PM‐MRI of the brain or abdomen that had additional information at autopsy. For the two cases with non‐diagnostic PM‐MRI of the heart that had autopsy findings, the autopsy was normal in one (50.0%) case and in the other there was a VSD.

## DISCUSSION

In this study we found that, when diagnostic images were obtained, there were no significant differences in accuracy between perinatal PM‐US and 1.5‐T PM‐MRI. If PM‐US were to be used as a frontline imaging tool instead of 1.5‐T PM‐MRI, the same overall diagnosis would be reached in the majority (> 85%) of cases. There was a higher rate of non‐diagnostic imaging on PM‐US compared with PM‐MRI, particularly of the brain and heart. Marked maceration was a common contributing factor to this for both modalities.

Our results are supported by the only other published work comparing PM‐US with PM‐MRI^8^. In that study, concordance with autopsy for final diagnosis was achieved in 67.8% (95% CI, 54.4–79.4%) of cases using PM‐US compared with in 78.0% (95% CI, 65.3–87.7%) of cases using 3‐T PM‐MRI (in our study these rates were 86.4% (95% CI, 77.7–92.0%) for PM‐US and 88.6% (95% CI, 80.3–93.7%) for 1.5‐T PM‐MRI). There were no statistical differences in their sensitivities and specificities between the two modalities for any of the five anatomical regions, as we also report. Although not significant, we found the largest differences in sensitivity between PM‐US and 1.5‐T PM‐MRI to be for cardiac (50.0% for PM‐US *vs* 81.8% for PM‐MRI) and thoracic (40.0% for PM‐US *vs* 73.3% PM‐MRI) abnormalities. For cardiac anomalies, the difference was mostly due to misses of complex cardiac anomalies for both modalities (but mostly PM‐US); for thoracic abnormalities it was due to misdiagnosis of subjective pulmonary hypoplasia. The low sensitivity of PM‐US could be explained by the lack of circulating blood, presence of intracardiac thrombus and gas, and the densely consolidated lungs in the postmortem state making diagnosis difficult.

With respect to non‐diagnostic studies, our results reflect closely those of Kang *et al*.^8^, who reported a higher non‐diagnostic rate for PM‐US than for 3‐T PM‐MRI, particularly for the brain (26.9% for PM‐US *vs* 4.4% for PM‐MRI (compared with 22.8% for PM‐US *vs* 3.7% for 1.5‐T PM‐MRI in our study)) and heart (30.6% for PM‐US *vs* 3.8% for 3‐T PM‐MRI (compared with 14.7% for PM‐US *vs* 5.1% for 1.5‐T PM‐MRI in our study)). In contrast to their results, we did not identify any non‐diagnostic abdominal or spinal examinations at PM‐US, whilst they reported non‐diagnostic rates of 23.7% and 1.9%, respectively. This could be due to differences in ultrasound systems, operator experience or interpretation of ‘diagnostic quality’. We found that marked maceration was a common factor in our non‐diagnostic cases. This information could be helpful when counseling parents about the potential success of a non‐invasive (imaging‐based) autopsy.

Our study has several clinical implications for the potential future role of PM‐US in perinatal non‐invasive autopsy, especially when access to PM‐MRI is limited or is unavailable. Given the high concordance for overall diagnosis between PM‐MRI and PM‐US, it is reasonable to suggest that PM‐US could be used as a first‐line or alternative imaging tool, particularly in cases in which an abdominal or spinal abnormality is suspected. Given that the lowest sensitivities and specificities were seen for cardiothoracic abnormalities, and that non‐diagnostic rates for brain and heart PM‐US were also high, PM‐MRI should be considered as the first‐line imaging tool for suspected cardiac malformations, and as a second‐line tool when PM‐US of the brain is non‐diagnostic. This would help to minimize missed diagnoses, given that autopsy confirmed the presence of intracranial and cardiac abnormalities in 50% and 25% of cases, respectively, when PM‐US was non‐diagnostic.

Our study has several limitations. The main one relates to our relatively small sample size, in particular the subset which also had autopsy results available, this group being smaller than intended according to our power calculation. This resulted in wide confidence intervals for many of our diagnostic‐accuracy rates, and may have precluded detection of any significant differences. Nevertheless, these early results show that the overall sensitivity and specificity rates for body organs and overall diagnoses for both imaging modalities were very similar to those of previously published work^6^, ^11^, ^20^, ^21^, ^22^, ^23^, ^24^, ^25^, ^26^, ^27^, ^28^, and we included as many cases from our center as possible, spanning a 5‐year study period. Second, we acknowledge that ultrasonography is operator‐dependent and our PM‐US was conducted by a specialist experienced pediatric radiologist at a tertiary center. It may be difficult to replicate this in other centers, and thus our diagnostic quality and accuracy rates may not be widely generalizable. We recommend comprehensive PM‐US training as appropriate before considering offering a PM‐US service to replace PM‐MRI. Finally, given the variation in the timing of imaging after fetal delivery or neonatal death, we cannot exclude the possibility that this may have contributed to the non‐diagnostic imaging quality or led to missed diagnoses. Given that our institution does not have an on‐site maternity unit, there is usually a delay in the processing and transport of cases. Similarly, the availability of our MRI scanner and radiologist is variable, replicating normal clinical practice. Performing imaging as close as possible to death or delivery may help improve diagnostic rates, although this remains to be established in larger studies.

In conclusion, this study has found high diagnostic accuracy rates for both PM‐US and 1.5‐T PM‐MRI, without significant differences between the two methods. If all cases undergoing 1.5‐T PM‐MRI were redirected to PM‐US imaging, the final diagnosis would be the same for the majority of cases. PM‐US could be implemented as a first‐line imaging tool in centers wishing to offer an affordable, non‐invasive autopsy service, with 1.5‐T PM‐MRI being most useful for suspected cardiac and brain malformations.

## Supporting information

**Table S1** Postmortem ultrasound (PM‐US) and postmortem magnetic resonance imaging (PM‐MRI) positive (LR+) and negative (LR–) likelihood ratios for individual body systems, all body systems summated and overall diagnoses, using autopsy as reference standard
**Table S2** Details of false‐negative (misses) and false‐positive (overcalls) diagnoses on postmortem ultrasound (PM‐US) and postmortem magnetic resonance imaging (PM‐MRI) (compared with autopsy data; *n* = 88)
**Table S3** Differences in overall diagnoses on postmortem ultrasound (PM‐US) compared with postmortem magnetic resonance imaging (PM‐MRI), in 136 fetuses which underwent perinatal death
**Table S4** Findings according to anatomical region, showing agreements and disagreements between postmortem ultrasound (PM‐US) and postmortem magnetic resonance imaging (PM‐MRI) findings overall, in 136 fetuses which underwent perinatal death

## References

[1] Godbole K, Bhide V, Nerune S, Kulkarni A, Moghe M, Kanade A. Role of fetal autopsy as a complementary tool to prenatal ultrasound. J Matern Fetal Neonatal Med 2014; 27: 1688–1692. 24313561 10.3109/14767058.2013.872094

[2] Nayak SS, Shukla A, Lewis L, Kadavigere R, Mathew M, Adiga PK, Vasudeva A, Kumar P, Shetty J, Shah H, Girisha KM. Clinical utility of fetal autopsy and its impact on genetic counseling. Prenat Diagn 2015; 35: 685–691. 25763538 10.1002/pd.4592

[3] Loughrey MB, McCluggage WG, Toner PG. The declining autopsy rate and clinicians' attitudes. Ulster Med J 2000; 69: 83–89. 11196736 PMC2449188

[4] Auger N, Bilodeau‐Bertrand M, Poissant J, Shah PS. Decreasing use of autopsy for stillbirths and infant deaths: missed opportunity. J Perinatol 2018; 38: 1414–1419. 30076403 10.1038/s41372-018-0191-y

[5] Heazell AE, McLaughlin MJ, Schmidt EB, Cox P, Flenady V, Khong TY, Downe . A difficult conversation? The views and experiences of parents and professionals on the consent process for perinatal postmortem after stillbirth. BJOG 2012; 119: 987–997. 22587524 10.1111/j.1471-0528.2012.03357.x

[6] Ashwin C, Hutchinson JC, Kang X, Langan D, Jones R, Norman W, Cannie M, Jani J, Sebire NJ, Arthurs OJ. Learning effect on perinatal post‐mortem magnetic resonance imaging reporting: single reporter diagnostic accuracy of 200 cases. Prenat Diagn 2017; 37: 566–574. 28342279 10.1002/pd.5043

[7] Arthurs OJ, Guy A, Thayyil S, Wade A, Jones R, Norman W, Scott R, Robertson NJ, Jacques TS, Chong WK, Gunny R, Saunders D, Olsen OE, Owens CM, Offiah AC, Chitty LS, Taylor AM, Sebire NJ; Magnetic Resonance Imaging Autopsy Study (MaRIAS) Collaborative Group . Comparison of diagnostic performance for perinatal and paediatric post‐mortem imaging: CT versus MRI. Eur Radiol 2016; 26: 2327–2336. 26489748 10.1007/s00330-015-4057-9

[8] Kang X, Cos Sanchez T, Arthurs OJ, Bevilacqua E, Cannie MM, Segers V, Lecomte S, Sebire NJ, Jani JC. Postmortem fetal imaging: prospective blinded comparison two‐dimensional ultrasound with magnetic resonance imaging. Ultrasound Obstet Gynecol 2019; 54: 791–799. 30644623 10.1002/uog.20217

[9] Jawad N, Sebire NJ, Wade A, Taylor AM, Chitty LS, Arthurs OJ. Body weight lower limits of fetal postmortem MRI at 1.5 T. Ultrasound Obstet Gynecol 2016; 48: 92–97. 26183321 10.1002/uog.14948

[10] Norman W, Jawad N, Jones R, Taylor AM, Arthurs OJ. Perinatal and paediatric post‐mortem magnetic resonance imaging (PMMR): sequences and technique. Br J Radiol 2016; 89: 20151028. 26916282 10.1259/bjr.20151028 PMC5258168

[11] Kang X, Shelmerdine SC, Hurtado I, Bevilacqua E, Hutchinson C, Mandalia U, Segers V, Cos Sanchez T, Cannie MM, Carlin A, Sebire NJ, Arthurs OJ, Jani JC. Postmortem examination of human fetuses: comparison of two‐dimensional ultrasound with invasive autopsy. Ultrasound Obstet Gynecol 2019; 53: 229–238. 28782198 10.1002/uog.18828

[12] Shelmerdine SC, Sebire NJ, Arthurs OJ. Perinatal post mortem ultrasound (PMUS): a practical approach. Insights imaging 2019; 10: 81. 31432284 10.1186/s13244-019-0762-2 PMC6702254

[13] The Royal College of Pathologists . Guidelines on autopsy practice: Third trimester antepartum and intrapartum stillbirth. June 2017. https://www.rcpath.org/uploads/assets/0e55a233‐5fe6‐4965‐b85f4fa7c9971742/Guidelines‐on‐autopsy‐practice‐Third‐trimester‐antepartum‐and‐intrapartum‐stillbirth.pdf.

[14] The Royal College of Pathologists . Guidelines on autopsy practice: Fetal autopsy (2^nd^ trimester fetal loss and termination of pregnancy for congenital anomaly). 2017. https://www.rcpath.org/uploads/assets/b20ea503‐7799‐433c‐99160653762f896c/Fetal‐autopsy‐2nd‐trimester‐fetal‐loss‐and‐termination‐of‐pregnancy‐for‐congenital‐anomaly.pdf.

[15] The Royal College of Pathologists . Guidelines on autopsy practice: Neonatal Death 2019. https://www.rcpath.org/uploads/assets/0a7c073e‐c773‐4941‐a1e998df666e17e3/G168‐Guidelines‐on‐autopsy‐practice‐Neonatal‐death.pdf.

[16] Sebire NJ, Weber MA, Thayyil S, Mushtaq I, Taylor A, Chitty LS. Minimally invasive perinatal autopsies using magnetic resonance imaging and endoscopic postmortem examination (“keyhole autopsy”): feasibility and initial experience. J Matern Fetal Neonatal Med 2012; 25: 513–518. 21740313 10.3109/14767058.2011.601368

[17] Hutchinson JC, Shelmerdine SC, Lewis C, Parmenter J, Simcock IC, Ward L, Ashworth MT, Chitty LS, Arthurs OJ, Sebire NJ. Minimally invasive perinatal and pediatric autopsy with laparoscopically assisted tissue sampling: feasibility and experience of the MinImAL procedure. Ultrasound Obstet Gynecol 2019; 54: 661–669. 30620444 10.1002/uog.20211

[18] Shelmerdine SC, Hutchinson JC, Ward L, Sekar T, Ashworth MT, Levine S, Sebire NJ, Arthurs OJ. Feasibility of INTACT (INcisionless TArgeted Core Tissue) biopsy procedure for perinatal autopsy. Ultrasound Obstet Gynecol 2019; 55: 667–675. 10.1002/uog.20387 PMC7317589 31271478

[19] Beam CA. Strategies for improving power in diagnostic radiology research. AJR Am J Roentgenol 1992; 159: 631–637. 1503041 10.2214/ajr.159.3.1503041

[20] Tuchtan L, Lesieur E, Bartoli C, Delteil C, Sarda‐Quarello L, Torrents J, Sigaudy S, Peircecchi MD, Gorincour G. Diagnosis of congenital abnormalities with post‐mortem ultrasound in perinatal death. Diagn Interv Imaging 2018; 99: 143–149. 29229509 10.1016/j.diii.2017.11.005

[21] Votino C, Cos Sanchez T, Bessieres B, Segers V, Kadhim H, Razavi F, Condorelli M, Votino R, D'Ambrosio V, Jani J. Minimally invasive fetal autopsy using ultrasound: a feasibility study. Ultrasound Obstet Gynecol 2018; 52: 776–783. 25130705 10.1002/uog.14642

[22] Prodhomme O, Baud C, Saguintaah M, Béchard‐Sevette N, Bolivar J, David S, Taleb‐Arrada I, Couture A. Comparison of postmortem ultrasound and X‐Ray with autopsy in fetal death: Retrospective study of 169 cases. JOFRI 2015; 3: 120–130.

[23] Ahmad MU, Sharif KA, Qayyum H, Ehsanullah B, Balyasnikova S, Wale A, Shanmuganandan A, Siddiqui MRS, Athanasiou T, Kemp GJ. Assessing the use of magnetic resonance imaging virtopsy as an alternative to autopsy: a systematic review and meta‐analysis. Postgrad Med J 2017; 93: 671–678. 28684530 10.1136/postgradmedj-2017-134945

[24] Arthurs OJ, Thayyil S, Addison S, Wade A, Jones R, Norman W, Scott R, Robertson NJ, Chitty LS, Taylor AM, Sebire NJ, Offiah AC; Magnetic Resonance Imaging Autopsy Study (MaRIAS) Collaborative Group . Diagnostic accuracy of postmortem MRI for musculoskeletal abnormalities in fetuses and children. Prenat Diagn 2014; 34: 1254–1261. 25043483 10.1002/pd.4460

[25] Arthurs OJ, Thayyil S, Olsen OE, Addison S, Wade A, Jones R, Norman W, Scott RJ, Robertson NJ, Taylor AM, Chitty LS, Sebire NJ, Owens CM; Magnetic Resonance Imaging Autopsy Study (MaRIAS) Collaborative Group . Diagnostic accuracy of post‐mortem MRI for thoracic abnormalities in fetuses and children. Eur Radiol 2014; 24: 2876–2884. 25173624 10.1007/s00330-014-3313-8 PMC4182596

[26] Arthurs OJ, Thayyil S, Pauliah SS, Jacques TS, Chong WK, Gunny R, Saunders D, Addison S, Lally P, Cady E, Jones R, Norman W, Scott R, Robertson NJ, Wade A, Chitty L, Taylor AM, Sebire NJ; Magnetic Resonance Imaging Autopsy Study (MaRIAS) Collaborative Group . Diagnostic accuracy and limitations of post‐mortem MRI for neurological abnormalities in fetuses and children. Clin Radiol 2015; 70: 872–880. 26050535 10.1016/j.crad.2015.04.008

[27] Arthurs OJ, Thayyil S, Owens CM, Olsen OE, Wade A, Addison S, Jones R, Norman W, Scott RJ, Robertson NJ, Taylor AM, Chitty LS, Sebire NJ; Magentic Resonance Imaging Autopsy Study (MaRIAS) Collaborative Group . Diagnostic accuracy of post mortem MRI for abdominal abnormalities in foetuses and children. Eur J Radiol 2015; 84: 474–481. 25533719 10.1016/j.ejrad.2014.11.030

[28] Arthurs OJ, Guy A, Thayyil S, Wade A, Jones R, Norman W, Scott R, Robertson NJ, Jacques TS, Chong WK, Gunny R, Saunders D, Olsen OE, Owens CM, Offiah AC, Chitty LS, Taylor AM, Sebire NJ; Magnetic Resonance Imaging Autopsy Study (MaRIAS) Collaborative Group . Comparison of diagnostic performance for perinatal and paediatric post‐mortem imaging: CT versus MRI. Eur Radiol 2016; 26: 2327–2336. 26489748 10.1007/s00330-015-4057-9

