# Supplementary material for: Diagnostic accuracy of postmortem ultrasound vs postmortem 1.5‐T MRI for non‐invasive perinatal autopsy
Source: Ultrasound Obstet Gynecol. 2021 Mar 1;57(3):449–58. doi: 10.1002/uog.22012 (PMC8432154; doi:10.1002/uog.22012)
Supplement: Supplementary file 1 — Table S1 Postmortem ultrasound (PM‐US) and postmortem magnetic resonance imaging (PM‐MRI) positive (LR+) and negative (LR–) likelihood ratios for individual body systems, all body systems summated and overall diagnoses, using autopsy as reference standard Table S2 Details of false‐negative (misses) and false‐positive (overcalls) diagnoses on postmortem ultrasound (PM‐US) and postmortem magnetic resonance imaging (PM‐MRI) (compared with autopsy data; n = 88) Table S3 Differences in overall diagnoses on postmortem ultrasound (PM‐US) compared with postmortem magnetic resonance imaging (PM‐MRI), in 136 fetuses which underwent perinatal death Table S4 Findings according to anatomical region, showing agreements and disagreements between postmortem ultrasound (PM‐US) and postmortem magnetic resonance imaging (PM‐MRI) findings overall, in 136 fetuses which underwent perinatal death [file UOG-57-449-s001.docx]

**Table S1** Postmortem ultrasound (PM-US) and postmortem magnetic resonance imaging (PM-MRI) positive and negative likelihood ratios (LR+, LR–) for individual body systems, all body systems summated and overall diagnoses, using autopsy as reference standard

|  | TP | FP | FN | TN | Sensitivity (%) | Specificity (%) | Concordance (%) | LR+ | LR– |
| --- | --- | --- | --- | --- | --- | --- | --- | --- | --- |
| Brain PM-US | 7 | 0 | 1 | 11 | 87.5  (52.9–97.8) | 100  (74.1–100) | 94.7  (75.4–99.1) | ∞ | 0.13  (0.02–0.78) |
| Brain PM-MRI | 10 | 0 | 2 | 14 | 83.3  (55.2–95.3) | 100  (78.5–100) | 92.3  (75.9–97.9) | ∞ | 0.17  (0.05–0.59) |
| Cardiac PM-US | 4 | 1 | 4 | 64 | 50.0  (21.5–78.5) | 98.5  (91.8–99.7) | 93.2  (84.9–97.0) | 32.5  (4.12–256.18) | 0.51  (0.25–1.02) |
| Cardiac PM-MRI | 9 | 1 | 2 | 69 | 81.8  (52.3–94.9) | 98.6  (92.3–99.7) | 96.3  (89.7–98.7) | 57.27  (8.02–408.96) | 0.18  (0.05–0.65) |
| Thoracic PM-US | 6 | 1 | 9 | 71 | 40.0  (19.8–64.3) | 98.6  (92.5–99.8) | 88.5  (80.1–93.6) | 28.8  (3.73–222.08) | 0.61  (0.40–0.92) |
| Thoracic PM-MRI | 11 | 3 | 4 | 68 | 73.3  (48.0–89.1) | 95.8  (88.3–98.6) | 91.9  (84.1–96.0) | 17.36  (5.50–54.74) | 0.28  (0.12–0.65) |
| Abdominal PM-US | 14 | 3 | 0 | 68 | 100  (78.5–100) | 95.8  (88.3–98.6) | 96.5  (90.1–98.8) | 23.67  (7.82–71.63) | 0.00 |
| Abdominal PM-MRI | 14 | 5 | 0 | 65 | 100  (78.5–100) | 92.9  (84.3–96.9) | 94.0  (86.8–97.4) | 14.00  (6.02–32.58) | 0.00 |
| Total body systems PM-US | 31 | 5 | 14 | 214 | 68.9  (54.3–80.5) | 97.7  (94.8–99.3) | 92.8  (89.0–95.3) | 30.17  (12.41–73.36) | 0.32  (0.21–0.49) |
| Total body systems PM-MRI | 44 | 9 | 8 | 216 | 84.6  (72.5–92.0) | 96.0  (92.6–97.9) | 93.9  (90.4–96.1) | 21.15  (11.04–40.54) | 0.16  (0.08–0.30) |
| Overall diagnosis* PM-US | 32 | 3 | 9 | 44 | 78.0  (63.3–88.0) | 93.6  (82.8–97.8) | 86.4  (77.7–92.0) | 12.23  (4.04–36.99) | 0.23  (0.13–0.42) |
| Overall diagnosis* PM-MRI | 37 | 6 | 4 | 41 | 90.2  (77.5–96.1) | 87.2  (74.8–94.0) | 88.6  (80.3–93.7) | 7.07  (3.33–15.03) | 0.11  (0.04–0.29) |

*Overall diagnosis refers to major pathology identified as cause of perinatal death.

**Table S2** Details of false-negative (misses) and false-positive (overcalls) diagnoses on postmortem ultrasound (PM-US) and postmortem magnetic resonance imaging (PM-MRI) (compared with autopsy data; *n* = 88)

|  | **False negatives (Misses)** | | | **False positives (Overcalls)** | | |
| --- | --- | --- | --- | --- | --- | --- |
|  | **Both PM-MRI + PM-US** | **PM-US only** | **PM-MRI only** | **Both PM-MRI + PM-US** | **PM-US only** | **PM-MRI only** |
| **Brain** | 1 x Severe hypoxic-ischemic encephalopathy, periventricular necrosis and extensive neuronal loss in the hindbrain | 0 | 1 x cerebellar hypoplasia | 0 | 0 | 0 |
| **Cardiac** | 1 x cardiomegaly (in the setting of CMV infection) | 1 x DCM  1 x DORV  1 x cardiac hypertrophy and dysplastic pulmonary valve. | 1 x VSD | 0 | 1 x VSD | 1 x DORV |
| **Thorax** | 1 x intra-alveolar hemorrhage  2 x pulmonary hypoplasis  1 x CMV infection | 1 x CLO  3 x pulmonary hypoplasia  1 x respiratory distress syndrome | 0 | 1 x pulmonary hypoplasia | 0 | 2 x pulmonary hypoplasia |
| **Abdomen** | 0 | 0 | 0 | 1 x anal atresia (anus present at autopsy)  1 x gas in hepatic veins suspicious for infection (no infection in abdomen) | 1 x pelvicalyceal dilatation in both kidneys | 1 x bowel perforation  1 x left renal hypoplasia  1 x right adrenal hemorrhage |

*CLO – congenital lobar overinflation; CMV – cytomegalovirus; DCM – dilated cardiomyopathy; DORV – double outlet right ventricle; VSD – ventricular septal defect*

|  | | **PM-US** | |
| --- | --- | --- | --- |
|  |  | **POSTIVE** | **NEGATIVE / Non-diagnostic** |
| **PM-MRI** | **POSITIVE** | ***n* = 46 cases**  **Neurological (*n* = 12):**  7 x ACC  2 x VM with intracranial hemorrhage  1 x VM with hydrops  1 x periventricular nodular heterotopia  1 x Holoprosencephaly  **Multisystem (*n* = 8):**  1 x Hydrops  1 x VACTERL  1 x vertebral anomalies + VM  1 x ACC + right renal hypoplasia  1 x bilateral renal agenesis + complex cardiac anomalies  1 x absent corpus callosum + hip dislocations + right MCDK  2 x head and neck teratomas + renal anomalies  **Renal (*n* = 9):**  2 x Bladder outlet obstruction  4 x ARPKD  1 x bilateral MCDK  1 x right MCDK , left PUJO  1 x solitary pelvic ectopic kidney  **Musculoskeletal (*n* = 6):**  2 x Thanatophoric dysplasia  2 x Osteogenesis imperfecta  1 x Dysplasia (type unspecified/unknown)  1 x Bilateral absent feet  **Thoracic (*n* = 4):**  3 x congenital diaphragmatic hernias  1 x Bilateral pulmonary hypoplasia  **Cardiac (*n* = 3):**  1 x VSD  1 x Tetralogy of Fallot  1 x Transposition of the great arteries with VSD  **Abdominal (*n* = 4):**  1 x hepatosplenomegaly (possible metabolic cause)  1 x splenomegaly (possible metabolic cause  1 x florid intra-abdominal gas (suggestive infection)  1 x exomphalos | ***n* = 16 cases**  **Neurological ( *n* = 10):**  2 x ACC  1 x ACC + sinus thrombosis  1 x ACC + limb anomalies  1 x ACC + occipital polymicrogyria  1 x extradural hematoma + periventricular hemorrhage  1 x Vein of Galen malformation + cardiac failure  2 x VM  1 x hemi-megalencephaly  **Renal (*n* = 1):**  1 x left renal hypoplasia  **Abdomen (*n* = 2):**  2 x gas in abdomen (possible sepsis or perforation)  **Cardiac (*n* = 2):**  1 x interrupted aortic arch + DORV  1 x Fallot’s type anomaly  **Thoracic (*n* = 1):**  1 x lung disease of prematurity |
|  | **NEGATIVE/**  **Non-diagnostic** | ***n* = 2 cases**  **Neurological (*n* = 1):**  1 x hypoplastic cerebellum  **Abdomen (*n* = 1):**  1 x pelvic right kidney | ***n* = 72 ‘normal’ cases** |

**Table S3** Differences in overall diagnoses at postmortem ultrasound (PM-US) compared with postmortem magnetic resonance imagine (PM-MRI) (*n* = 136)

Whilst none of the studies were non-diagnostic for all body areas, some were non-diagnostic for certain body parts. *ACC – absent corpus callosum; ARPKD – autosomal recessive polycystic kidney disease; MCKD – multicystic dysplastic kidney disease; PUJO – pelviureteric junction obstruction; VM – ventriculomegaly; VSD – ventricular septal defect*

|  | **PM-MRI POSITIVE** | | | **PM-MRI NEGATIVE** | **PM-MRI NON-DIAGNOSTIC** |
| --- | --- | --- | --- | --- | --- |
|  | **PM-US POSITIVE** | **PM-US NEGATIVE** | **PM-US ND/NE** | **PM-US POSITIVE** | **PM-US POSITIVE** |
| **Brain** | 7 x ACC  1 x ACC + microcephaly  1 x ACC + VM  1 x ACC + VM + PVNH  1 x Teratoma  4 x VM  2 x VM + intracranial hemorrhage  1 x VM + PVNH | 1 x hemimegalencephaly  4 x VM  4 x ACC  1 x ACC + sinus thrombosis  1 x mega cisterna magna + small cerebellum  1 x cystic hygroma | 1 x ACC  1 x ACC + polymicrogyria  1 x intracranial hemorrhage  1 x Vein of Galen malformation | 1 x mild VM  1 x cerebellar hypoplasia  1 x ACC | 0 |
| **Cardiac** | 1 x Fallot’s type anomaly  1 x large VSD  1 x TGA + VSD  1 x dilated pulmonary artery with ASD | 1 x cardiomegaly  1 x Fallot’s type anomaly  1 x biventricular hypertrophy | 1 x TGA  1 x DORV  1 x HLHS | 0 | 1 x VSD |
| **Thoracic** | 3 x pulmonary hypoplasia  4 x congenital diagphragmatic hernia | 6 x pulmonary hypoplasia  1 x lung disease of prematurity | 0 | 0 | 0 |
| **Abdominal** | 6 x bilateral multicystic kidneys  2 x splenomegaly  1 x anal atresia  1 x large bladder, right renal cyst  1 x bilateral renal agenesis  1 x right renal agenesis  1 x right renal hypoplasia  1 x hepatosplenomegaly  1 x gas in hepatic system (possible infection)  1 x solitary pelvic kidney  1 x bladder outlet obstruction  1 x exomphalos  1 x right MCDK, left PUJO | 1 x heterogenous hepatic + renal appearances.  2 x locules of intra-abdominal gas  1 x renal pelvicalyceal dilatation.  1 x left renal hypoplasia  1 x right adrenal hemorrhage | 0 | 1 x renal pelvicalyceal dilatation | 1 x pelvic right kidney |
| **Spine** | 0 | 0 | 0 | 1 x thinning of the spinal cord | 0 |

**Table S4** Findings according to anatomical region, showing agreements and disagreements between post-mortem ultrasound (PM-US) and MRI (PM-MRI) findings overall (*n* = 136)

Cases in which both imaging modalities were negative for pathology, or in which one was negative and the other non-diagnostic/not examined, are not included herein, given no pathology to describe. *ACC – absent corpus callosum; ASD – atrial septal defect; DORV – double outlet right ventricle; HLHS – hypoplastic left heart syndrome; MCKD – multicystic dysplastic kidney disease; PUJO – pelviureteric junction obstruction; PVNH – periventricular nodular heterotopia; TGA – transposition of the great arteries; VM – ventriculomegaly; VSD – ventricular septal defect.*
